# Prevalence of Oral Mucosal Diseases in Older Adults in Mainland China: A Meta-Analysis of Observational Studies

**DOI:** 10.3390/ijerph17061887

**Published:** 2020-03-14

**Authors:** Na Zhou, Xin Zhang, Jia-Qing Yan, Ya-Qin Yu, Yan Cai

**Affiliations:** 1 Hospital of Stomatology, Jilin University, Changchun 130021, China; zhoun708@jlu.edu.cn (N.Z.); yjq@mail.jlu.edu.cn (J.-Q.Y.); 2 Department of Pharmacy, The First Hospital of Jilin University, Changchun 130021, China; happy_potato531@163.com; 3 Department of Epidemiology and Biostatistics, School of Public Health, Jilin University, Changchun 130021, China; yuyaqin5540@163.com

**Keywords:** oral mucosal disease, older adults, meta-analysis, prevalence

## Abstract

Oral mucosal disease (OMD) is a public health challenge globally, but the epidemiological findings in older adults have been inconsistent in China. Thus, this meta-analysis was carried out to explore the prevalence of OMD and its moderating factors in this population. An electronic literature search was conducted of both international (PubMed, PsycINFO, and EMBASE) and Chinese (China National Knowledge Infrastructure and WanFang) databases from inception to November 1, 2019. The Der–Simonian and Laird random effects model was used to synthesize the prevalence of OMD and its 95% confidence intervals (95% CI). Twenty-four studies covering 23,653 older adults were included. The pooled prevalence of OMD was 23% (95% confidence interval: 17.9%–29.0%) Subgroup analyses and meta-analysis revealed that the prevalence of OMD was significantly associated with the reporting sampling, year of publication, and survey (all *p* values <0.05). This meta-analysis found that the prevalence of OMD among older adults in mainland China was significantly high. Early detection and effective intervention of OMD in older adults have public health and clinical importance.

## 1. Introduction

In China, the percentage of people in China aged 60 years or over is rising dramatically [1]; persons of increasing age are more likely to suffer from various oral problems [2]. At present, though a certain proportion of oral mucosal diseases (OMD) does not need active treatment, but there is some evidence that people with oral diseases are associated with various negative outcomes, such as poor quality of life, a heavy global burden on social and economic health, high risk of disability, and impaired physical function [3,4], particular in the elderly [5,6].

The prevalence of OMD in older adults in China ranges from 0.0% to 83.8% across studies [7,8]. For example, a study from Jiangxi province did not find any OMD patients in the older adult population [8]; however, in a study on the older adults in an urban community of Xinjiang Uygur Autonomous Region, the prevalence of OMD was 83.8%, higher in men (87.01%) than in women (75%) [7]. Another study on the oral health survey of Sichuan province between 2015 and 2016 found that 8.2% of participants aged 65–74 years had OMDs [9]. The mixed findings may be partly owing to the differences in survey time across studies, as well as in ethnic background, behavior, and lifestyles across the populations. To achieve a reasonable allocation of health resources, the right policy development, implement effective preventive measures and treatments, and significantly reduce of health outcomes of OMD in older adults, better comprehension of the OMD pattern is necessary.

To the best of our knowledge, no meta-analysis or systematic review on the prevalence of OMD in older adults has been published so far. Hence, we carried out a systematic review and meta-analysis of epidemiological studies to explore the prevalence and moderating factors (i.e., the sources of heterogeneity) of OMD in older adults. Following the previous findings from observational studies [9,10], we hypothesize that the prevalence of OMD in mainland China is relatively high in older adults to date.

## 2. Methods

### 2.1. Data Sources and Search Strategies

The meta-analysis followed the Preferred Reporting Items for Systematic Reviews and Meta-analyses (PRISMA) checklist. Two investigators (N.Z. and X.Z.) extensively searched online international (PubMed, EMBASE, PsycINFO) and Chinese (Chinese National Knowledge Infrastructure, WanFang) databases from inception to 1 November 2019. The search terms were MeSH terms and text words linked to oral mucosal disease (salivary Gland Diseases OR xerostomia OR oral mucosal diseases OR Sjogren’s syndrome OR hyposalivation OR Asialia OR Asialias OR mouth dryness OR Dryness, Mouth), epidemiology (epidemiology OR cross-sectional OR prevalence OR rate), old people (old* OR elderly), China (China OR Chinese).

### 2.2. Study Eligibility

Two investigators (N.Z. and X.Z.) independently screened all titles and abstracts from the initial search results, as well as full-text articles identified from the first-stage screening (titles and abstracts), with discrepancies resolved through discussion or consultation via a senior investigator (Y.C.). The references of the included studies were additionally reviewed in order to collect any potential studies.

To meet analysis requirements and reduce selection bias, articles were eligible if (a) older adults aged ≥60 years, (b) cross-sectional or retrospective surveys, and (c) prevalence of OMD with or without providing relevant data were reported. Studies excluded (a) reviews, case reports, protocols, comments, (b) single disease from OMD, or (c) special populations (such as militants).

### 2.3. Data Extraction

The data extracted independently by two investigators (N.Z. and X.Z.), including study characteristics (e.g., first author, publication year, province, sample size), participant characteristics (e.g., mean age or age range, gender), main outcome (events or prevalence rate with corresponding 95% CI). Any disagreements were resolved by a discussion, consensus, or consulting another researcher (Y.C.).

### 2.4. Quality Assessment

Parker’s quality evaluation tool for prevalence studies was used to evaluate the methodological quality of the included studies [11]. The included studies were assessed by the definition and representativeness of the targeted population, sampling methods, response rate, the definition of the target symptom or diagnosis, and validation of the assessment instrument. Each item was considered as “1 (yes)” or “0 (no or unclear)”. Discrepancies were resolved by consensus with a third author (Y.C.).

### 2.5. Statistical Analyses

Due to the anticipated substantial heterogeneity, the random-effects model was utilized to calculate the prevalence of OMD with 95% CI. Heterogeneity across studies was tested by I^2^ and Q statistics (I^2^ >50% was regarded as significant heterogeneity; Higgins and Thompson, 2002 [12]). Publication bias was assessed by visual inspection of the funnel plots, Begg’s and Egger’s tests. Subgroup analyses were stratified by publication language (English or Chinese), sampling (yes or no), and region according to the National Bureau of Statistics of China (west vs. east vs. middle). Year of publication, survey year based on end year, sample size, the proportion of males, and study quality were analyzed by meta-regression analyses based on unrestricted maximum likelihood in order to detect the main sources of heterogeneity [13]. Comprehensive Meta-Analysis Program version 2.0 (Biostat Inc., Englewood, NJ, USA) was used with a significant level of 0.05 (two-sided).

## 3. Results

### 3.1. Study Selection and Characteristics

**Figure 1:**
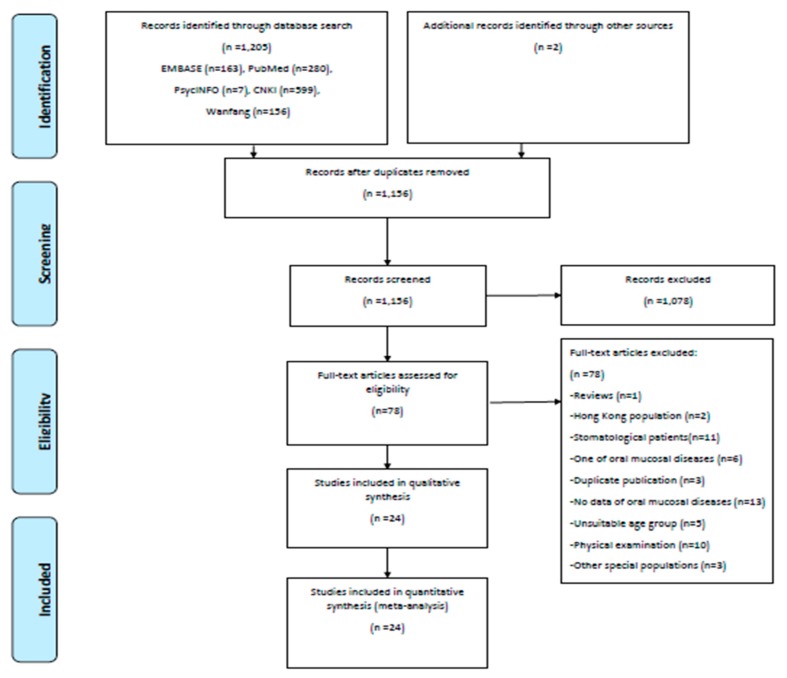
Flowchart of literature selection.

A total of 24 articles fulfilling our review criteria were identified (Figure 1). Overall, the included studies were published between 1985 and 2018, involving 15 provinces: 14 studies in eastern China, 3 in central China, 7 in western China. The sample sizes in the included study ranged from 50 to 3349 participants, with a median sample size of 733. The detailed study characteristics and outcomes are presented in Table 1.

### 3.2. Prevalence of Oral Mucosal Diseases, Subgroup and Meta-Regression Analyses

**Figure 2:**
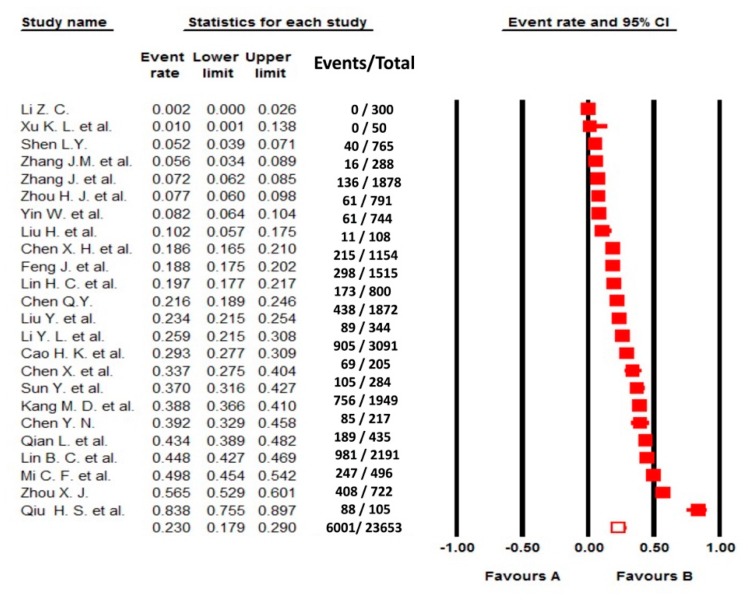
Forest plot of the prevalence of oral mucosal diseases in older adults.

The pooled OMD prevalence among older adults was 23% (n = 6001; 95% CI: 17.9%–29.0%), with a significant heterogeneity (I^2^ = 98.87%; Figure 2). Subgroup analyses only found that pooled OMD prevalence in the reporting sampling method group (16.6%) was lower than that in the non-reporting sampling method group (31.0%; Table 2). Additionally, the meta-regression analysis found year of publication and survey significantly associated with the prevalence of OMD (both *p* values < 0.05; Table 3).

### 3.3. Quality Assessment and Publication Bias

**Figure 3:**
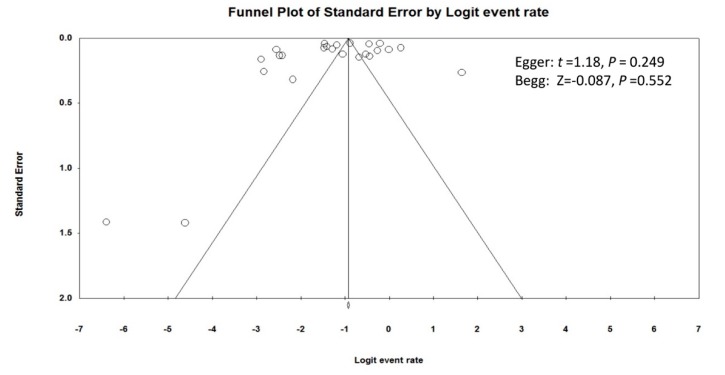
Funnel plot of publication bias.

The median quality assessment score of the 24 studies was 5, ranging from 2 to 6. The Egger’s and Begg’s tests did not identify publication bias (Egger: *t* = 1.18, *p* = 0.249; Begg: Z = −0.087, *p* = 0.552), with a symmetrical funnel plot (Figure 3).

## 4. Discussion

To the best of our knowledge, this is the first meta-analysis to examine the prevalence of OMD in older adults in mainland China. The pooled prevalence of OMD was 23% (95% CI: 17.9%–29.0%) in older adults. A Chinese report of the development on aging reported that there were approximately 202 million old people in 2013 [34], which would equate to nearly 46.46 million old people experiencing OMD based on the current results. Common OMD could be due to several reasons. First, older adults may not have formed good oral health-related behaviors when they were young due to lack of financial resources, oral health awareness, and family oral education. Second, having difficulty getting about and memory decline are very common in the older population. Thus, maintaining oral health is not easy in the long term.

To date, some studies have examined the epidemiology of OMD, but the prevalence rates are inconsistent in older adults across studies. The prevalence of OMD in older adults aged 60 and above is relatively high, ranging from 29.0% in Iran, over 33.3% in Australia, 41.2% in the USA, to 53% in Chile [35,36,37,38,39]. The pooled prevalence of OMD in this meta-analysis is significantly lower than the corresponding figures (29%–53%) reported from most of the Western countries, but not all (2%–3% in South Africa) [37]. The discrepancy of the OMD rate between Chinese and Western studies in older adults could be due to several reasons. On the one hand, this could partly be due to different sampling methods, definitions of OMD, or local clinical practice and guidelines. On the other hand, oral health may be viewed as a small matter in China and it is not recorded if subjects are not seriously affected in daily life, resulting in an underestimation of OMD.

Subgroup analyses indicated that the prevalence of OMD was higher in studies with no reporting sampling method (31.0% vs. 16.6%), which could be the fact that the findings of studies without reporting sampling method are relatively more unstable [40]. In addition, the findings from meta-regression revealed that the year of publication and survey were two moderating factors in the prevalence of OMD. One important reason is that with increasing attention on oral health and the implementation of some relevant health policies in China [41], people gradually began to maintain oral hygiene and prevent oral problems. Thus, studies conducted in recent years show lower prevalence rates.

Several limitations should be noted in this meta-analysis. First, similar to other meta-analyses of epidemiological studies [42,43], significant heterogeneity was identified, although random-effect models were carried out. The source of heterogeneity may result from some unreported factors, such as different ethnicities and comorbidity status (e.g., diabetes, hypertension, and hyperlipidemia). Second, the 24 studies involved only 15 out of 31 provinces, autonomous regions, and municipalities of mainland China, which restricts the generalizability of the findings. Third, the included articles were limited to only English and Chinese languages; thus, the findings may be biased. Fourth, a relatively small number of English papers was included. However, the Chinese population is our concern, and Chinese people get used to publishing Chinese papers in Chinese journals. Fifth, the prevalence of OMD by gender and region has not been reported because of the limited number of studies. Nevertheless, meta-regression did not find that the proportion of males and the proportion of urban dwellers would significantly impact the results. Lastly, important factors related to OMD, such as sub-classification, economic status, family background, and use of medicine, were not analyzed due to insufficient data.

In summary, our findings suggest that the prevalence of OMD in older adults is common in mainland China. Given the high prevalence of OMD in this population, screening and intervention for underlying OMD have significance in clinical settings and public health regarding OMD prevention and treatment. In addition, in order to reduce the high prevalence of OMD, oral knowledge should be strengthened, oral education should be delivered, and oral monitoring should be regularly conducted for the older population. Finally, longitudinal research about the associations between OMD and other demographic and clinical variables in the older population should be conducted in the future.

## Figures and Tables

**Table 1:** Characteristics of the studies included in the meta-analysis.

No.	First Author	Publication Year	Publication Language	Survey Year	Sampling	Sample Size	Male (N, %)	Urban (N, %)	Category of OMD	Number of OMD	Age (Mean ± SD)	Age Range	Province	Area	Quality Score	References
1	Kang M. D. et al.	1985	CH	NR	No	1949	754 (38.7)	1531 (78.6)	WH, PM and others	756	NR	60–104	Henan	M	2	[14]
2	Xu K. L. et al.	1985	CH	1983	No	50	12 (24.0)	34 (68.0)	NR	0	92.4 ± NR	90–101	Guangxi	E	2	[15]
3	Chen Y. N.	1987	CH	1982	No	217	121 (55.8)	217 (100)	EM, LK, WH, PM and others	85	NR	60–87	Zhejiang	E	3	[16]
4	Sun Y. et al.	1987	CH	NR	No	284	263 (92.6)	284 (100)	LP, WE, CG, XS and others	105	NR	≥60	Liaoning	E	2	[17]
5	Cao H. K. et al.	1988	CH	1986	No	3091	1230 (39.8)	3091 (100)	FT, KA, AG, RAU and others	905	NR	≥60	Shanghai	E	3	[18]
6	Chen X. et al.	1989	CH	1987	Yes	205	145 (70.7)	205 (100)	OS, FK, WE, FT and others	69	NR	≥60	Ningxia	W	5	[19]
7	Lin B. C. et al.	1989	CH	NR	Yes	2191	1086 (49.6)	2004 (91.5)	OS, FK, WE, FT and others	981	NR	≥60	Beijing	E	6	[20]
8	Mi C. F. et al.	1991	CH	1989	No	496	391 (78.8)	496 (100)	LK, LP, RAU, XS, OS	247	NR	≥60	Ningxia	W	2	[21]
9	Shen L.Y.	1995	CH	1994	No	765	649 (84.8)	765 (100)	LK, LP and others	40	NR	60–75	Jiangsu	E	2	[22]
10	Li Y. L. et al.	1996	CH	1994–1995	Yes	344	256 (69.8)	344 (100)	MH, LP, RAU and others	89	67.48 ± NR	60–77	Inner Mongolia	M	5	[23]
11	Qiu H. S. et al.	1996	CH	NR	No	105	77 (73.3)	0 (0)	FT, LS, MP and others	88	NR	100–135	Xinjiang	W	4	[7]
12	Zhou X. J.	2000	CH	NR	No	722	NR	769 (100)	OS, FT, WE and others	408	NR	≥60	Beijing	E	3	[24]
13	Lin H. C. et al.	2001	EN	1997	Yes	1515	759 (50.1)	774 (51.1)	LP, RAU, FT and others	298	NR	65–74	Guangdong	E	6	[25]
14	Liu Y. et al.	2001	CH	1999	No	1872	785 (41.9)	1872 (100)	FT, WE, LK, LP and others	438	67.8 ± NR	60–91	Chongqing	E	4	[26]
15	Chen Q.Y.	2006	CH	NR	No	800	400 (50.0)	NR	RAU, LP, LK and others	173	NR	60–95	Jiangsu	E	2	[27]
16	Chen X. H. et al.	2007	CH	2003	Yes	1154	519 (45.0)	1154 (100)	KA, LP, LK and others	215	NR	≥60	Guangdong	E	5	[28]
17	Liu H. et al.	2009	CH	2007	No	108	54 (50.0)	NR	NR	11	NR	65–70	Xinjiang	W	5	[29]
18	Zhou H. J. et al.	2009	CH	2005	Yes	791	391 (49.4)	419 (53.0)	LK, LP and others	61	68.0 ± NR	65–74	Gansu	W	5	[30]
19	Qian L. et al.	2011	CH	2009–2011	Yes	435	135 (31.0)	435 (100)	OC, SS, FT, KA and others	189	79.22 ± NR	≥60	Jiangsu	E	5	[31]
20	Feng J. et al.	2015	EN	2012–2013	Yes	3349	NR	3349 (100)	LK, LP, FT and others	630	NR	≥60	Shanghai	E	5	[10]
21	Zhang J. et al.	2016	CH	2015–2016	Yes	1878	895 (47.7)	NR	LK, LP, and others	136	NR	65–74	Ningxia	W	5	[32]
22	Yin W. et al.	2017	EN	2015–2016	Yes	744	362 (48.7)	365 (49.1)	FT, RAU, LP and others	61	NR	65–74	Sichuan	W	6	[9]
23	Li Z. C.	2017	CH	2015–2016	Yes	300	150 (50.0)	150 (50.0)	NR	0	NR	65–74	Jiangxi	M	6	[8]
24	Zhang J.M. et al.	2018	CH	2015–2016	Yes	288	144 (50.0)	NR	LK, LP and others	16	NR	65–74	Guangdong	E	5	[33]

OMD = oral mucosal diseases; WH = white hyperkeratosis; PM = pigmentation; EM = erythema; LK = leukoplakia; KA = keratosis albicans; AG = atrophic glossitis; RAU = recurrent aphthous ulcer; OS = oral smoke spots; OC = oral candidiasis; FK = friction keratosis; WE = white edema; LP = lichen planus; CG = chronic glossitis; XS = xerostomia syndrome; LK = leukoplakia; MH = mucosal hyperkeratosis; MP = mucosal plaque; FT = fissured tongue; SS = Sjogren’s syndrome; CH = Chinese; EN = English; NR = not Reported; SD = standard deviation; E = East area; M = Middle area; W = West area.

**Table 2:** Subgroup analyses.

Category	Variables	Classification	Sample Size	Effect Size	95% CI		I^2^	*p* Across Subgroup
**Subgroup analysis**	Region	East area (3)	16,733	0.237	0.170	0.320	98.82	0.944
		Middle area (4)	2593	0.209	0.087	0.421	94.70	
		West area (1)	4327	0.222	0.137	0.338	99.13	
	Publication language	EN (3)	5608	0.147	0.071	0.281	95.97	0.155
		CN (21)	18,045	0.247	0.191	0.312	98.78	
	Sampling	Yes (12)	13,194	0.166	0.113	0.237	99.05	0.011
		No (12)	10,459	0.310	0.223	0.413	98.41	

CH = Chinese; EN = English.

**Table 3:** Meta-regression analyses.

Category	Variables	Slope	S.E.	95% CI		*t*	*p*
Meta-regression	Year of publication	−0.060	0.019	−0.096	−0.024	−3.24	0.001
	Survey year	−0.047	0.018	−0.082	−0.013	−2.69	0.007
	Sample size	0.0001	0.0003	−0.0004	0.0006	0.53	0.599
	Study quality	−0.187	0.190	−0.559	0.184	−0.99	0.323
	Proportion of male	0.020	0.016	−0.011	0.052	1.28	0.199
	Proportion of urban	0.002	0.010	−0.019	0.022	0.15	0.880

S.E.= Standard Error; CI= Confidence Interval

## References

[1] World Health Organization (2016). China Country Assessment Report on Ageing and Health. https://apps.who.int/iris/bitstream/handle/10665/194271/9789241509312_eng.pdf?sequence=1.

[2] Petersen P.E., Yamamoto T. (2005). Improving the oral health of older people: The approach of the WHO Global Oral Health Programme. Community Dent. Oral Epidemiol..

[3] Petersen P.E. (2009). Global policy for improvement of oral health in the 21st century–implications to oral health research of World Health Assembly 2007, World Health Organization. Community Dent. Oral Epidemiol..

[4] Jin L., Lamster I., Greenspan J., Pitts N., Scully C., Warnakulasuriya S. (2016). Global burden of oral diseases: Emerging concepts, management and interplay with systemic health. Oral Dis..

[5] Kotronia E., Wannamethee G., Papacosta O., Whincup P., Lennon L., Visser M., Weyant R., Harris T., Ramsay S. (2019). Oral health, disability and physical function: Results from studies of older people in the UK and USA. J. Epidemiol. Community Health.

[6] Fattori E., Teixeira D.S., de Figueiredo M.A.Z., Cherubini K., Salum F.G. (2019). Stomatological disorders in older people: An epidemiological study in the Brazil southern. Med. Oral Patol. Oral Cir. Bucal..

[7] Qiu H.S., Li C.F., feng M.M., Bai S.Y., Wang D.R. (1996). A survey of oral mucosal status in the 105 Uighur centenarian in Xinjiang. J. Pract. Stomatol..

[8] Li Z.C. (2017). Oral Health Survey and Influencing Factors Analysis among Adult in Jiangxi Province. Ph.D. Thesis.

[9] Yin W., Yang Y.M., Chen H., Li X., Wang Z., Cheng L., Yin Q.D., Fang H.Z., Fei W., Mi F.L. (2017). Oral health status in Sichuan Province: Findings from the oral health survey of Sichuan, 2015-2016. Int. J. Oral Sci..

[10] Feng J., Zhou Z., Shen X., Wang Y., Shi L., Wang Y., Hu Y., Sun H., Liu W. (2015). Prevalence and distribution of oral mucosal lesions: A cross-sectional study in Shanghai, China. J. Oral Pathol. Med..

[11] Parker G., Beresford B., Clarke S., Gridley K., Pitman R., Spiers G., Light K. (2008). Technical Report for SCIE Research Review on the Prevalence and Incidence of Parental Mental Health Problems and the Detection, Screening and Reporting of Parental Mental Health Problems. Ph.D. Thesis.

[12] Higgins J.P., Thompson S.G. (2002). Quantifying Heterogeneity in a Meta-Analysis. Stat. Med..

[13] Knapp G., Hartung J. (2003). Improved tests for a random effects meta-regression with a single covariate. Stat. Med..

[14] Kang M.D., Zhang Z.L., Zhang B.H., Niu Y.P., Hu Y.Z., han F.Z., Huang J.H., Su Z.Y., Li Y.Q. (1985). A survey of oral maxillofacial health in elderly people in Zhengzhou. Cent. Plains Med. J..

[15] Xu K.L., Hu S.D. (1985). A survey of oral status in 50 elderly people aged 90 years and above in Guilin. J. Clin. Stomatol..

[16] Chen Y.N. (1987). A survey of oral health in 2171 elderly inhabitants in Hangzhou. Zhejiang Med. J..

[17] Sun Y., Chen Y.X., Bai S.Y., Bai Z.N., Yang S., Han J., Li Z., Hu S.F. (1987). A survey of oral health in 284 elderly people in Jinzhou. J. Jinzhou Med. Coll..

[18] Cao H.K., Le F.Y., Chen J.H., Lu H., Shi H.B., Shen Z.Y., Hu H.F., Jin W.C., L.Y. H., Tang G.Y. (1988). A survey of oral mucosal diseases in 3091 elderly inhabitants in Shanghai. Stomatology.

[19] Chen X., Han Y.A., Wang C., Huang S.J., Li Z.Q., Wang Z.F., Li N.F. (1989). A survey of oral mucosal diseases in Yinchuan. J. Ningxia Med. Coll..

[20] Lin B.C., Dai Y.Y., Zhu Y.X., Zhang R.D. (1989). A survey of oral mucosal diseases in 2191 elderly inhabitants in Beijing. Chin. J. Stomatol..

[21] Mi C.F., He T. (1991). A survey of oral diseases in 496 elderly people in Yinchuan. Ningxia Med. J..

[22] Shen L.Y. (1995). A survey of oral health in 765 elderly people in Jiangsu Province. Stomatology.

[23] Li Y.R., Xu Y.D., Xun H., Zhang T.J. (1996). A survey of oral health status of 344 elderly people in Yakeshi, Inner Mongolia. J. Mod. Stomatol..

[24] Zhou X.J. (2000). A survey of oral health in 769 elderly people. J. Clin. Stomatol..

[25] Lin H.C., Corbet E.F., Lo E.C.M. (2001). Oral mucosal lesions in adult Chinese. J. Dent. Res..

[26] Liu Y., Wang L. (2001). A survey of oral health in 1872 elderly people in Chongqing. J. Mod. Stomatol..

[27] Chen Q.Y. (2006). A survey of oral health in elderly people. J. Med. Theory Pract..

[28] Chen X.H., Zhu A.D. (2007). A survey of oral mucosal health in 1154 elderly inhabitants in Guangzhou. J. Dent. Prev. Treat..

[29] Liu H., Zhong L.J., Mamat Y., Li D., Jiang H., Zhao X.G., Delixiat Y. (2009). A survey of oral health status of the older Uighur people in hetian, Xinjiang. Chin. J. Geriatr. Dent..

[30] Zhou H.J., Nie H.Y., Yang L., Ma L.Y., Li Z.Q., Nie H.B., Feng Z.H. (2009). A survey of oral mucosal diseases in middle-aged and elderly people in Gansu Province. Chin. J. Public Health.

[31] Qian L., Wu G.Y., Li L.Y., Lu L., Jiang J.M., Shi Y., Li N., Zhang K., Qi H.J., Liu L.W. (2011). Survey on oral mucosal health of the aged people in the apartments for the elderly in urban of Nanjing. Oral Biomed..

[32] Zhang J., Li D., Li M.Y., Sha J.J., Jiang X.J. (2016). Prevalence of oral mucosal diseases and related risk factors in the middle aged and elderly people of Hui and Han population in Zhongwei City, Ningxia Province. Chin. J. Conserv. Dent..

[33] Zhang J.M., Li J.B., Fang W.H., Zhao W.H., Liu Z.Q., Huang S.H. (2018). A sample survey report on oral mucosa status of middle-aged and elderly people in Guangdong province from 2015 to 2017. China Med. Pharm..

[34] Wu Y., Dang J. (2013). Blue Book of Aging: China Report of the Development on Aging Cause (2013).

[35] Mansour Ghanaei F., Joukar F., Rabiei M., Dadashzadeh A., Kord Valeshabad A. (2013). Prevalence of oral mucosal lesions in an adult Iranian population. Iran Red. Crescent Med. J..

[36] Do L., Spencer A., Dost F., Farah C. (2014). Oral mucosal lesions: Findings from the Australian National Survey of Adult Oral Health. Aust. Dent. J..

[37] Pontes C.C., Chikte U., Kimmie-Dhansay F., Erasmus R.T., Kengne A.P., Matsha T.E. (2020). Prevalence of Oral Mucosal Lesions and Relation to Serum Cotinine Levels—Findings from a Cross-Sectional Study in South Africa. Int. J. Environ. Res. Public Health.

[38] Espinoza I., Rojas R., Aranda W., Gamonal J. (2003). Prevalence of oral mucosal lesions in elderly people in Santiago, Chile. J. Oral Pathol. Med..

[39] Shulman J.D., Beach M.M., Rivera-Hidalgo F. (2004). The prevalence of oral mucosal lesions in US adults: Data from the Third National Health and Nutrition Examination Survey, 1988–1994. J. Am. Dent. Assoc..

[40] Martínez-Mesa J., González-Chica D.A., Duquia R.P., Bonamigo R.R., Bastos J.L. (2016). Sampling: How to select participants in my research study?. Bras Derm..

[41] Liu J., Zhang S.S., Zheng S.G., Xu T., Si Y. (2016). Oral health status and oral health care model in China. Chin. J. Dent. Res..

[42] Rao W.-W., Zeng L.-N., Zhang J.-W., Zong Q.-Q., An F.-R., Ng C.H., Ungvari G.S., Yang F.-Y., Zhang J., Peng K.Z. (2019). Worldwide prevalence of falls in older adults with psychiatric disorders: A meta-analysis of observational studies. Psychiatry Res..

[43] Qi H., Zong Q.-Q., Lok G.K.I., Rao W.-W., An F.-R., Ungvari G.S., Balbuena L., Zhang Q.-E., Xiang Y.-T. (2019). Treatment Rate for Major Depressive Disorder in China: A Meta-Analysis of Epidemiological Studies. Psychiatr. Q..

